# Bio-Fabrication of *Euryale ferox* (Makhana) Leaf Silver Nanoparticles and Their Antibacterial, Antioxidant and Cytotoxic Potential

**DOI:** 10.3390/plants11202766

**Published:** 2022-10-19

**Authors:** Nisha Devi, Kanika Rani, Pushpa Kharb, Prashant Kaushik

**Affiliations:** 1 Department of Molecular Biology, Biotechnology and Bioinformatics, Chaudhary Charan Singh Haryana Agricultural University, Hisar 125004, India; 2 Center for Bio-Nanotechnology, Chaudhary Charan Singh Haryana Agricultural University, Hisar 125004, India; 3 Kikugawa Research Station, Yokohama Ueki, Kikugawa 439-0031, Japan

**Keywords:** *Euryale* *ferox*, leaf extract, silver nanoparticles, antibacterial, makhana, cytotoxicity, antioxidant activity

## Abstract

Bio-fabrication of green or plant extract-based silver nanoparticles has garnered much praise over the past decade as the methodology is environment-friendly, undemanding, non-pathogenic, and economical. In the current study, leaves of *Eurale ferox* (Makhana), considered as waste, were used for the bio-fabrication of silver nanoparticles (ELAgNPs). Various analytical techniques including UV–VIS spectroscopy, Field emission scanning electron microscopy equipped with an energy dispersive X-ray spectrometer (FESEM-EDX), Particle size analyzer (PSA), Fourier transform infra-red spectroscopy (FTIR) and high-resolution transmission electron microscopy (HRTEM) were used for their characterization. Their antibacterial efficacy was examined against gram positive bacterium, *Bacillus subtilis* and gram negative bacterium, *Escherichia coli*. The antioxidant potential of the ELAgNPs was compassed by 2, 2 diphenyl-1-picryl hydrazyl (DPPH; λ_max_ = 517 nm) assay, H_2_O_2_ (λ_max_ = 230 nm) and OH^−^ (λ_max_ = 520 nm)-based radical scavenging assays. The cytotoxicity was checked against the VERO cell line using 3-[4, 5-dimethyl thiazol-2-yl]-2, 5 diphenyl tetrazolium bromide (MTT) assay. A mean particle size of 26.51 ± 8.87 nm with a size distribution of 7.08–53.94 nm was obtained using HRTEM. The ELAgNPs exhibited dose-dependent antibacterial efficacy with a maximum zone of inhibition (ZOI) of 21.98 ± 0.59 mm against *B. subtilis* and of 16.46 ± 0.22 mm against *E. coli* at 500 ppm after 24 h of incubation. The median lethal concentration for the cytotoxicity analysis was found to be 9.54 ± 0.35 ppm, 120.9 ± 6.31 ppm, and 20.74 ± 0.63 ppm for ELAgNPs, commercial silver nanoparticles (CAgNPs), and silver nitrate (SN), respectively. The ordinary one-way ANOVA results exhibited a significant decrease in cell viability after 72 h of incubation at *p* < 0.05, α = 0.05. In conclusion, the ELAgNPs showed good antibacterial, radical scavenging and dose-dependent cytotoxicity against the VERO cells. Therefore, these could be used for biomedical applications. Phyto-constituents present in the plant not only act as reducing agents but also as stabilizing and coating agents, and the availability of a wide range of metabolites makes the green approach more promising.

## 1. Introduction

Nanotechnology is one of the paramount technologies of the modern era that has the potential to revolutionize the day-to-day aspects of human life. Nanomaterials include nanoparticles, nanocomposites, nanofibers, nanotubes, etc., having a minimum of one dimension within the range of 1–100 nm. They exhibit peculiar physicochemical, electrical, optical, and magnetic properties based on their shape, size and distribution compared with their bulk counterparts [1]. Therefore, they have multifarious applications in various disciplines of science, such as electronics, optics, chemistry, energy, biotechnology, medicine, agriculture, and cleaning technology. They could be employed in targeted drug delivery, wastewater treatment, and in the production of nanofertilizers, nanopesticides, and biosensors [2]. 

Two approaches are used for the production of nanoparticles (NPs), top-down and bottom-up. In top-down approaches, the bulk material is reduced to nanoscale, whereas in the bottom-up approaches, the starting material (atoms/molecules) assembles to form nanomaterial. Top-down approaches include physical methods like ball milling, evaporation–condensation, arc discharge, laser ablation, and spray pyrolysis. In contrast, bottom-up approaches include chemical methods (chemically vapor-based deposition, sol-gel, reverse micelle, and liquid state synthetic approaches) and biological methods. Metallic NPs can be synthesized using various physical, chemical, and biological approaches. However, the requirement for large amounts of energy, a substandard yield and complex scheming for instruments are a few of the shortcomings linked to physical approaches [3], and chemical methods employ vaporous and unsafe compounds that are hazardous to the environment [4]. In biological methods, entities like algae, fungi, bacteria, and plants are used for eco-friendly synthesis. Major drawbacks associated with the microbes-based biological methods are the maintenance of microbial cultures, labor- and time-intensive processes, and biohazards. However, the plant-based biological or green synthesis overcomes these problems as the usage of plants is cost-effective, economical, non-pathogenic and environment-friendly [5]. 

Silver nanoparticles (AgNPs) have great potential among various metallic NPs. They exhibit a wide array of applications, including antimicrobial, cytotoxic, anticancerous agents, virucidal [6] and in cardio protection, wound dressing, and cosmetics [7]. Their properties are strongly dependent on the conditions of the synthesis process, such as type of reaction, precursor, and reagents used, type of equipment used, concentration, temperature, duration, and pH. The use of plant extracts to produce AgNPs appears to be more convenient, as the methodology is simple, environment-friendly, and inexpensive [8]. Phyto-constituents present in plants not only act as reducing agents but also as stabilizing and coating agents. Moreover, the availability of a wide range of metabolites, makes the green approach more promising [9,10]. 

*E. ferox* Salisb., commonly known as Fox Nut or Gorgon Nut (in English) and Makhana (in Hindi), belongs to the Nymphaeaceae family; ‘*ferox*’ is the sole species within the ‘*Euryale*’ genus in this family. Makhana is an aquatic plant with massive buoyant leaves and is widely dispersed over Asian countries like Japan, Korea, India, and China [11]. It grows as a rich harvest in the depthless aquatic systems (shallows) of two states in India, namely, Assam and Bihar. It prefers tropical and subtropical climates, including a temperature ranging from 20 °C to 35 °C, a downpour of 100 cm to 250 cm, and high humidity (50% to 90%). Makhana seeds have been used in Ayurvedic and Chinese medicine for ages and are called Black Diamond due to their immense therapeutic applications [12], including antidiabetic [13], antihyperlipidemic, hepatoprotective [14], antimelanogenic [15], anticancerous [16], antiaging [17], cardio protection [18], and antidepressant [19].

Several studies have been conducted to explore the medicinal potential of Makhana seeds, but only a very few reports are available for the pharmaceutical usage of leaves as they have been considered biowaste until now. In Chinese medicine, they are used in dystocia [20], in the preparation of vinegar [21], and in the golden flower concept for the generation of probiotic fungus, *Eurotium cristatum* [22]; they could also have radical scavenging property as being rich in anthocyanins since their abaxial surface is dark purple [23]. Anthocyanins are a type of flavonoid which generally occur as glycosides of flavylium salts differing in –OH and –OCH_3_ substitutions of aromatic ring B. Different types of anthocyanins, including delphinidin, pelargonidin, cyanidin, malvidin, peonidin, and petunidin with strong antioxidant potential, were identified using HPLC-QTOF-MS/MS in leaves of Makhana [24].

In this study, we present the synthesis of AgNPs using a hot hydrothermal leaf extract of Makhana (ELAgNPs) in a fast and robust way. To the best of our knowledge, this is the first report on the bio-fabrication of AgNPs using Makhana leaves. The antibacterial, antioxidant and cytotoxic activities of these particles have also been investigated. 

## 2. Methodology and Material Required

### 2.1. Material Required

Garden-fresh leaf tissue of *E. ferox* was collected from plants grown at Botanical Park, CCS HAU, Hisar, India. The pathogenic bacteria *B. subtilis* (Gram positive) and *E. coli* (Gram negative) were obtained from the Department of Veterinary Microbiology, LLRUVAS, Hisar, India. For the cytotoxicity analysis, African green monkey kidney cells, VERO, were procured from the Department of Animal Biotechnology, LLRUVAS, Hisar, India. All the chemicals, namely commercial AgNPs (CAgNPs), nutrient agar (NA), AgNO_3_ (SN), Tetrazolium salt (MTT), H_2_O_2_, buffers, ascorbic acid, picrylhydrazyl salt (DPPH), etc., were of cell-culture and analytical grade (Sigma-Aldrich^®^ Chemical Co., St. Louis, MO, U.S.). The purchased CAgNPs were coated with polyvinylpyrrolidone (PVP) and were below 100 nm in size with spherical morphology as exhibited in the TEM analysis (provided in product specification).

### 2.2. Green Synthesis of ELAgNPs

**Figure 1:**
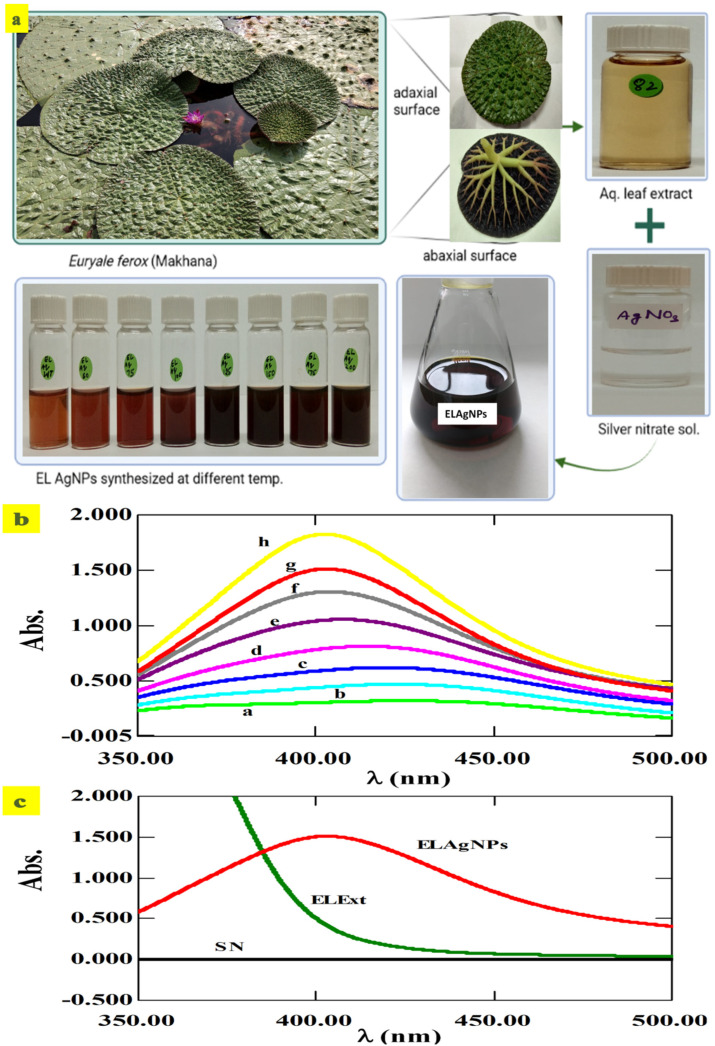
Synthesis of aqueous leaf extract of *E. ferox*-based AgNPs (ELAgNPs). (**a**) *E. ferox* leaf morphology and bioreduction of Ag⁺ ions in silver nitrate solution (SN) by hydrothermal *E. ferox* leaf extract (ELExt) at different temperatures, (**b**) UV–VIS spectral peaks of synthesized ELAgNPs at various temperatures (where, a = 23 ± 2 °C, b = 50 °C, c = 75 °C, d = 100 °C, e = 125 °C, f = 150 °C, g = 175 °C, and h = 200 °C), and (**c**) absorption spectra of ELExt, ELAgNPs, and SN at 175 °C.

Freshly harvested leaves were thoroughly washed with tap water (3–4 times) to remove dirt, then 2–3 times with distilled water, and then finally rinsed with deionized water (Figure 1a). Finely ground 5 g leaves were added to deionized water (100 mL) and boiled for 10 min. This was further centrifuged at 10k rpm for a duration of 10 min. Separated supernatant was filtered using a syringe filter of 0.22 μm and then stored in a sterilized reagent bottle at 4 °C for further use. Hydrothermal leaf extract (ELExt) was added to 1 mM AgNO_3_ solution (SN) at a ratio of 1:20 at varying temperatures, including room temperature (23 ± 2) °C, 50 °C, 75 °C, 100 °C, 125 °C, 150 °C, 175 °C and 200 °C on a magnetic stirrer (Figure 1a). Absorption spectra were reported using a UV–Visible spectrophotometer (UV-2600, Shimadzu, Kyoto, Japan) for confirmation of the synthesis of ELAgNPs. The SPR peaks of the freshly synthesized colloidal ELAgNP solution were recorded at a wavelength of 350–500 nm where AgNO_3_ was used as blank.

### 2.3. Characterization of ELAgNPs

The characterization of synthesized NPs is critical for understanding their morphology, distribution, behavior and functional aspects. The colloidal stability via zeta potential (ZP) and the distribution of the particle size of the ELAgNPs were determined by PSA (Nanotrac Wave II, Metrohm, Chennai, India) at 10kV field strength. The infra-red spectrum was recorded with an FTIR spectrometer (Nicolet iS50 FTIR, Thermo Fisher Scientific, Waltham, MA, USA) for the identification of functional groups involved in their bioreduction, capping and stabilization at λ ranging from 4000 cm^−1^ to 400 cm^−1^. The surface morphology of the ELAgNPs was investigated utilizing FESEM (NanoSEM 450, Thermo-Fisher Scientific, USA) at an accelerated voltage of 15 kV with an Everhart-Thornley (ETD) detector. The lyophilized sample was first gold coated and then analyzed. Their elemental composition was measured by an EDX spectrometer (QUANTAX, Bruker Corporation, Billerica, MA, USA) equipped with SEM. Moisture-free lyophilized the ELAgNPs were coated upon a carbon film for the EDX experiment. The size and shape of the ELAgNPs were confirmed by HRTEM (Tecnai G2, Thermo Fisher Scientific, USA) having an LaB6 filament at a point resolution of 0.2 nm with 200 kV accelerating voltage. For the TEM analysis, a drop of ELAgNP solution was placed on a carbon-coated Cu- grid and then dried. After that, the measurement was taken.

### 2.4. Antibacterial Activity of ELAgNPs

The agar well diffusion method was used for antibacterial efficacy against the pathogenic bacteria *B. subtilis* and *E. coli* [25]. Fresh overnight cultures with an OD of 0.5 McFarland (approximately 1–2 × 10^8^ CFU/mL) were used as inoculums and seeded on NA plates by a sterile cotton swab. The surface of the agar medium plate was bored by sterile tips to make wells of 6 mm. A quantity of 100 μL of each concentration (1, 10, 50, 100, 250, and 500 ppm) of synthesized ELAgNPs with four replications were used. Various controls including ELExt, SN, CAgNPs and media control (NA only) were also used. These sample plates along with the control were incubated at 37 °C. After 12 and 24 h of incubation, the zone of inhibition (ZOI) was measured.

### 2.5. Antioxidant Potential of ELAgNPs

#### 2.5.1. DPPH-Based Antioxidant Activity

One ml of different concentrations (1, 10, 50, 100, 250, and 500 ppm) of the ELAgNPs was added to 1 mL of stable radical DPPH (1 mM in CH_3_OH) solution in reference to standard vitamin C and vortexed thoroughly [26]. Further, the reaction mixture was incubated at 23 ± 2 °C for 30–35 min in the dark. The optical density (OD) of the ELAgNPs and the control was observed at λ_max_ = 517 nm by a spectrophotometer (CH_3_OH utilized as blank). The antioxidant efficacy (%) was expressed as below:

DPPH antioxidant activity (%) = [{OD_X_ – OD_Y/Z_ }/OD_X_] × 100 (1)

where X = stable radical DPPH as control, Y = ELAgNPs, Z = vitamin C.

#### 2.5.2. Hydrogen Peroxide Scavenging Activity 

A quantity of 100 μL of various concentrations (1, 10, 50, 100, 250, and 500 ppm) of the ELAgNPs were mixed with 300 μL of phosphate buffer and 600 μL of 2 mM H_2_O_2_ solution (prepared in phosphate buffer) [27] and shaken to mix. The OD at λ_max_= 230 nm was measured for the ELAgNPs, standard Vitamin C and the control H_2_O_2_ solution (phosphate buffer utilized as blank) after 15 min of reaction. The concentration of blank was 50 mM with pH of 7.4. The H_2_O_2_ scavenging activity (%) was calculated as below:

(2) $$\%\;\mathrm{of}\;\mathrm{scavenging}=\frac{\left\{\mathrm{OD}\;\mathrm{of}\;H_{2}O_{2}-\left(\mathrm{OD}\;\mathrm{of}\;\frac{\mathrm{ELAgNPs}}{\mathrm{Vitamin}\;C}\right)\right\}}{\mathrm{OD}\;\mathrm{of}\;H_{2}O_{2}}\times 100$$

#### 2.5.3. OH^−^ Radical Scavenging Activity

A quantity of 70 μL of various concentrations (1, 10, 50, 100, 250, and 500 ppm) of the ELAgNPs were added to 445 μL of 200 mM NaH_2_PO_4_ buffer (pH = 7.2), 150 μL of 10 mM of each (deoxyribose, FeSO_4_-EDTA H_2_O_2_, and 535 μL of deionized water [27]) and the reaction mixture was placed in an incubator for 4 h. The end point of the chemical reaction was achieved after the incorporation of 750 μL of each of 1% thiobarbituric acid and 2.8% trichloroacetic acid. Further, this solution was kept in water bath for 10–12 min at 80–95 °C. The OD was measured at λ_max_ = 520 nm after cooling down the reaction mixture along with standard Vitamin C and blank methanol. The OH^−^ radical scavenging activity (%) was expressed as below:

% of OH^−^ Radical Scavenging = [{OD_A_ – OD_B/C_}/OD_A_] × 100 (3)

where A = Mixture of all the reagents except ELAgNPs utilized as control, B = ELAgNPs, C = vitamin C.

### 2.6. Toxicity Analysis of ELAgNPs

The toxicity analysis of the ELAgNPs was assessed against the VERO cell line, which was grown and maintained in an autoclaved minimum essential medium (MEM), C_5_H_10_N_2_O_3_ (L-Gln), fetal bovine serum (10%), 4-(2-hydroxyl ethyl)-1-piperazine ethane-sulfonic acid (HEPES), antibiotics (100 ppm streptomycin, 100 I.U. penicillin), and sodium carbonate. The cell line was maintained by a routine subculture in 2% MEM and a culture with 75–85% confluency was used for the toxicity analysis.

For the cytotoxicity analysis, 100 μL of cell aliquot was seeded (per well) in a 96 well plate with a cell density of 2 × 10^4^ cells/ ml. A quantity of 100 μL of different concentrations of ELAgNPs (1.0, 2.5, 5.0, 10.0, 25.0, 50.0, 100.0, 250.0, and 500.0 μg mL^−1^ or ppm) were added to each well after an incubation period of 24 h along with the controls of ELExt, SN and CAgNPs. Cells were kept in a CO_2_ incubator for an additional period of 48 h at 37 °C. Then, 20 μL of tetrazolium salt ‘MTT’ prepared in PBS with a concentration of 5 mg/ mL was added to each well. Further, incubation of 4 h was provided to these MTT treated cells. After aspiration of the reagents in the plate, di-methyl sulfoxide (100 μL) was added to each well for the dissolution of purple colored crystals of formazan. An ELISA plate reader (Bio TeK EPOCH, China) was used for OD measurement at 570 nm [28]. The percentage of cell viability was determined as below:

Cell viability (%) = [OD of ELAgNPs)/ OD of experimental control × 100(4)

The median lethal concentration (LC_50_) was calculated for various concentrations of ELAgNPs, CAgNPs, ELExt and SN using GraphPad Prism version 9 software (GraphPad company, San Diego, CA, USA).

### 2.7. Statistical Analysis

Antibacterial activity in terms of the ZOI was tested in quadruplicate and the mean values were calculated. The antioxidant and scavenging potential of the ELAgNPs were tested in triplicates and the mean values were calculated. The LC_50_ of the ELAgNPs was subjected to the analysis of variance (ANOVA) using GraphPad Prism version 9. 

## 3. Results and Discussion

### 3.1. Green Synthesis and Characterization of ELAgNPs

Synthesis of reddish-brown to blackish-brown colored ELAgNPs by the bioreduction of Ag⁺ in AgNO_3_ solution by hydrothermal leaf extract at varying temperatures was confirmed from UV–VIS spectroscopy. The SPR peaks of the ELAgNPs obtained during UV–VIS analysis can reflect their size and shape. The broad bell-shaped surface plasmon resonance (SPR) peaks (‘a’ to ‘h’) of 424 nm, 423 nm, 419 nm, 414 nm, 407 nm, 403 nm, 403 nm, and 402 nm at an absorbance of 0.32, 0.68, 0.62, 0.81, 1.05, 1.31, 1.51, and 1.83 were obtained at (23 ± 2) °C, 50 °C, 75 °C, 100 °C, 125 °C, 150 °C, 175 °C, and 200 °C, respectively, and indicated their small size as shown in Figure 1b (a–h curves). The sharp narrow SPR peaks and high absorbance obtained at 150 °C, 175 °C, and 200 °C (‘f’ to ‘h’) pointed towards the smaller size of the ELAgNPs. The broadened peaks from ‘a’ to ‘e’ that shifted towards a longer wavelength indicated either the larger size or the agglomeration of the synthesized particles. The bioreduction could be due to phyto-constituents (anthocyanins, sugars, carboxylic acids) present in the aqueous leaf extract of Makhana as these plant metabolites participate in redox reactions and help in the synthesis and stabilization of AgNPs [29]. Our results are in agreement with the methanolic root extract of *Rhazya stricta*-based AgNPs showing a UV absorbance peak at 402 nm [30] and with hydrothermal fruit extract of *Areca catechu*-based AgNPs showing broadened peaks at a lower temperature and concentration [31].

**Figure 2:**
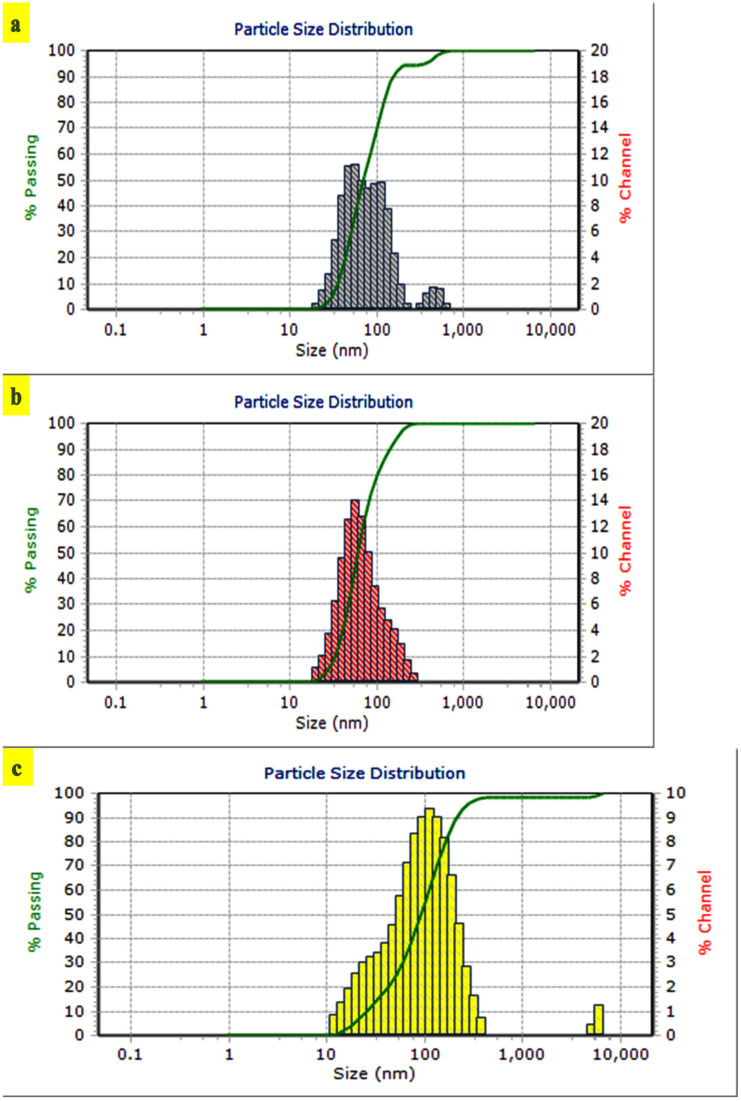

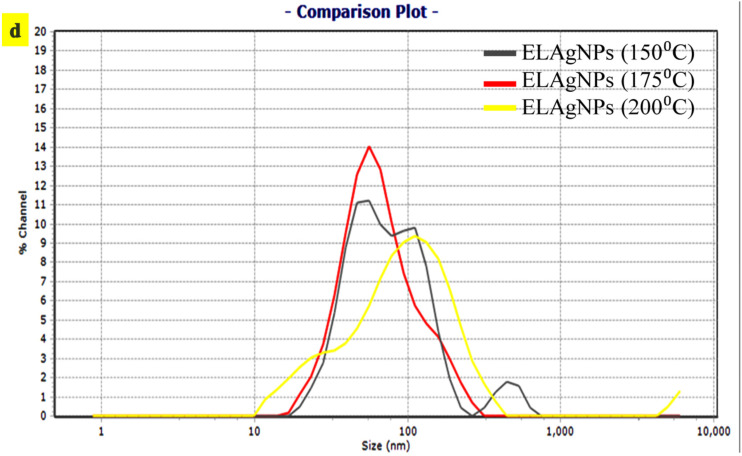
Particle size distribution of aqueous leaf extract of *E. ferox*-based AgNPs (ELAgNPs) (**a**) at 150 °C, (**b**) at 175 °C, and (**c**) at 200 °C. (**d**) Comparison plot of average hydrodynamic diameter (nm) of ELAgNPs synthesized at different temperatures on a stirrer.

Further, the stability and mono-dispersity of the ELAgNPs synthesized at different temperatures were confirmed by PSA. Particles synthesized at room temperature to 125 °C were highly unstable as exhibited by the larger polydispersity index of 0.63 to 0.81 and the larger average hydrodynamic diameter ranging from 263 nm to 6000 nm. Meanwhile, the ELAgNPs synthesized at 150 °C exhibited an average hydrodynamic diameter of 47.9 nm, 107 nm, and 453 nm with a volume % of 51.1%, 43.4%, and 5.5%, respectively, with a polydispersity index of 0.26 and negative ZP of −25.2 mV with very low reflectance power (Figure 2a). The ELAgNPs synthesized at 200 °C exhibited an average hydrodynamic diameter of 91.3 nm and 5800 nm with a volume % of 98.2% and 1.8%, respectively, with a polydispersity index of 0.213 and negative ZP of −28.5 mV with very low reflectance power (Figure 2c). In contrast to these, the ELAgNPs synthesized at 175 °C exhibited a mean diameter of 61.1 nm with the lowest polydispersity index of 0.13 confirming their mono-dispersity. Moreover, the highly negative zeta potential of −22.5 mV indicated their stability in preventing their agglomeration by repelling each other (Figure 2b). This was further confirmed by the comparison plot analysis of the average hydrodynamic diameter of these three formulations indicating a uniform mono-dispersive stable particle size distribution of the ELAgNPs synthesized at 175 °C with a sharp peak among the three (Figure 2d). These were selected for further characterization and bioefficacy studies. The obtained zeta potential results are in agreement with prior research reports exhibiting the negative zeta potential of −21.7 mV [32] and of −20 to −24 mV [33] of stable AgNPs synthesized using different plant extracts.

**Figure 3:**
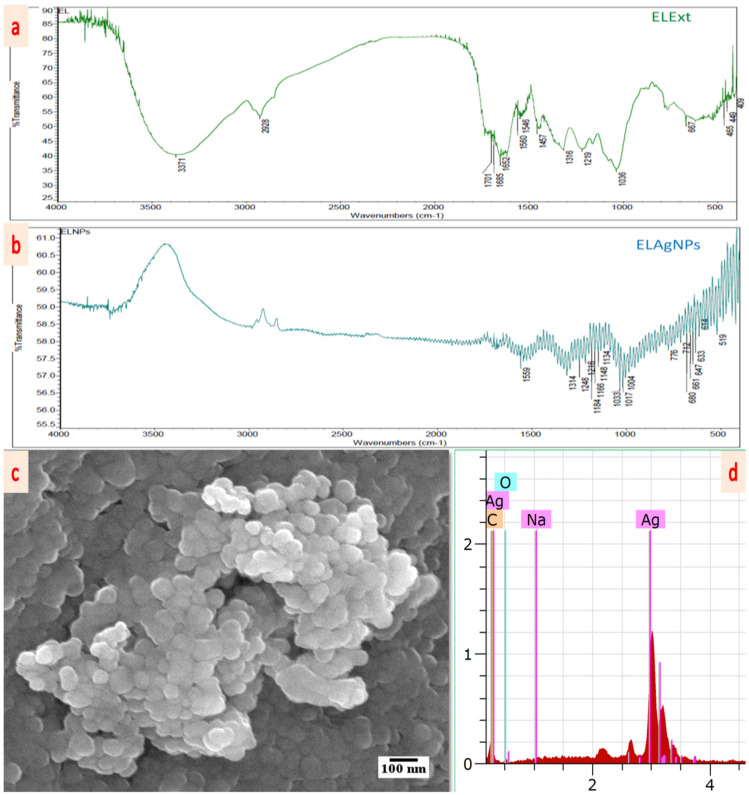
FTIR spectrum of (**a**) ELExt and (**b**) ELAgNPs. (**c**) FESEM micrographs of ELAgNPs. (**d**) EDX spectrum of ELAgNPs.

The marginal shift in FTIR spectral peaks of ELExt was seen during the synthesis of ELAgNPs indicating the presence of some remnant entities of phyto-constituents of the extract on the surface of the synthesized ELAgNPs as depicted in Figure 3a,b. *E. ferox* leaf powder exhibited peaks at 3371, 2928, 1701, 1685, 1652, 1560, 1546, 1457, 1316, 1219, and 1036 cm^−1^ (Figure 3a). The ELAgNPs exhibited spectral peaks at 3735, 1559, 1314, 1248, 1216, 1186, 1167, 1148, 1134, 1053, 1033, 1017, and 1004 cm^−1^ (Figure 3b). FTIR investigation revealed that bioactive compounds present in ELExt, including anthocyanins, glycosides, amines, carboxylic acids, aromatic compounds, etc., could be coated around the ELAgNPs for their steadiness and restriction to their aggregation [34]. Moreover, phyto-constituents present in the leaf extract acted as powerful reducing agents for the synthesis of ELAgNPs. The peaks from 1559–1596 cm^−1^ might be assigned to carbonyl phytocompounds. The sharp peaks from 1300–1420 cm^−1^ represent -C– N- stretching as well as an amide-I band of proteins present in the extract [35]. The presence of 1248 and 1216 cm^−1^ peaks are related to tertiary amines and in-plane -C-H bending of aromatic compounds present in ELExt. The intense peaks from 950–1250 cm^−1^, 1130–1190 cm^−1^, and from 1100–1200 cm^−1^ could be attributed to the presence of aromatic compounds, secondary amines, and sulphonates, respectively [36]. The sharp peaks from 1003–1095 cm^−1^ are assigned to phosphate ion, which is an important component of nucleic acids, ATP, proteins, lipids, etcetera [35].

**Figure 4:**
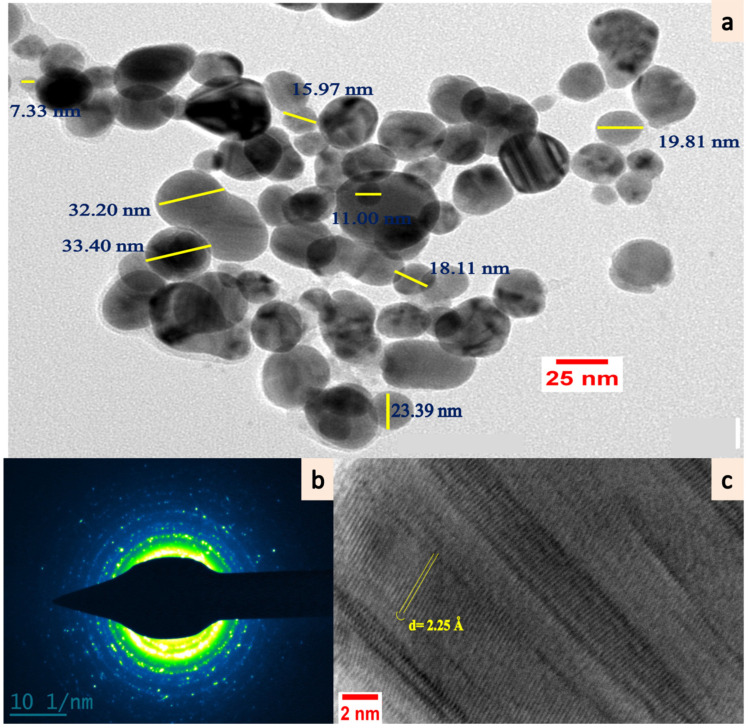
HRTEM micrographs of (**a**) synthesized ELAgNPs, (**b**) SAED pattern of crystalline ELAgNPs, and (**c**) single spherical ELAgNP with d-spacing.

The SEM micrographs showed that the particles were oval or spherical with a layered arrangement (Figure 3c) and the EDX spectrum revealed a strong characteristic peak of metallic AgNPs at 3 keV (Figure 3d). The quantitative outcome of the EDX spectrum also exhibited an abundance of 93.62 (weight%) of elemental Ag in the L-series of X-rays with the highest purity. Further, detailing of the shape and size of synthesized AgNPs was provided by HRTEM and the particles were spherical in shape and morphology (Figure 4a). The mean particle size obtained was 26.51 ± 8.87 nm with a particle size distribution of 7.08–53.94 nm. The polycrystalline behavior of the mono-dispersed ELAgNPs was evident from the selected area electron diffraction (SAED) rings generated by HRTEM showing clear concentric rings of metallic AgNPs (Figure 4b). The d-spacing (lattice-spacing) between atomic planes was found to be 2.25 angstrom (Figure 4c). The obtained SEM-EDX and TEM findings were in agreement with the reports [37,38] showing spherical mono-dispersive AgNPs synthesized using aqueous rhizome extracts of *Acorus calamus* and *Phlomis* spp. leaf extract, respectively. 

### 3.2. Assessment of Antibacterial Efficacy of ELAgNPs

**Figure 5:**
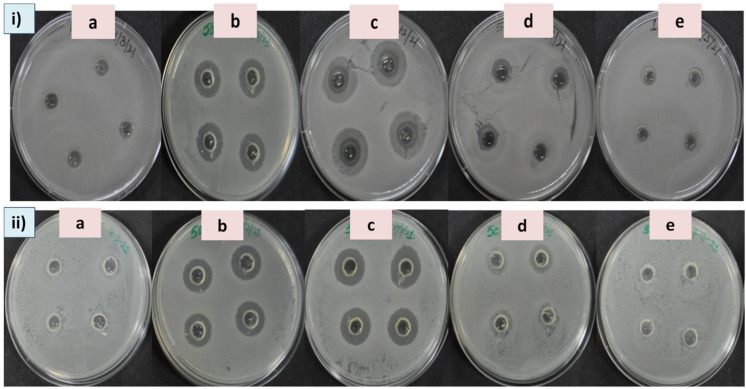
Zone of inhibition after 24 h of incubation at 500 ppm in (**i**) *B. subtilis*: (a) media control, (b) SN, (c) ELAgNPs, (d) CAgNPs, and (e) ELExt; and (**ii**) in *E. coli*: (a) media control, (b) SN, (c) ELAgNPs, (d) CAgNPs, and (e) ELExt.

**Figure 6:**
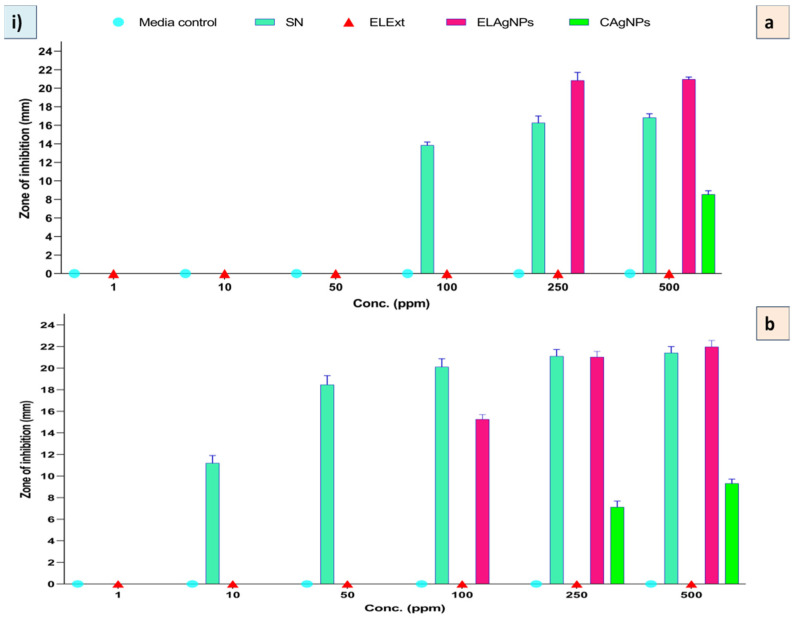

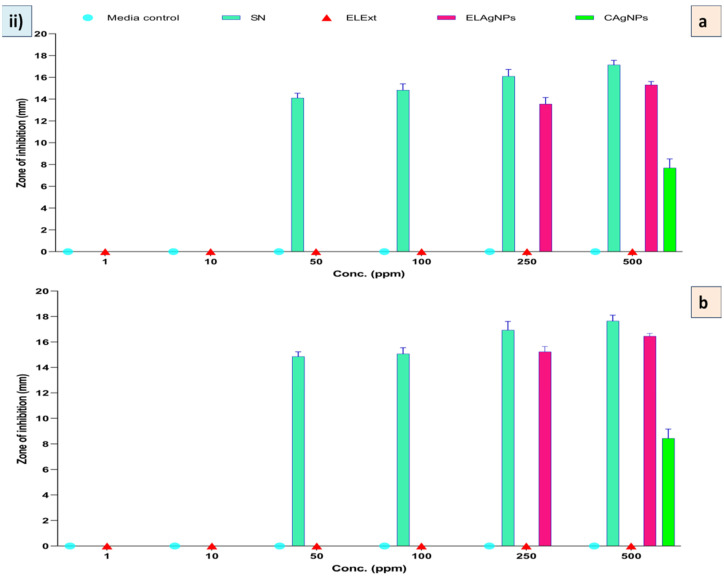
Antibacterial activity of green synthesized ELAgNPs against (**i**) *B. subtilis* and (**ii**) *E. coli* after (a) 12 h and (b) 24 h of incubation (data are presented as mean ± SD of four replicates).

Multidrug resistance in pathogenic bacteria has recently become a serious problem [39]. AgNPs are widely used in many clinical applications as they possess broad spectrum antibacterial properties. Therefore, the biosynthesized ELAgNPs were used as antibacterial agents against pathogenic bacteria. It was observed that ELExt and CAgNPs failed to inhibit bacterial growth at all tested concentrations after 12 and 24 h of incubation in both the targets. However, the ELAgNPs along with SN exhibited differential antimicrobial activity against the tested microorganisms. After 12 h of incubation, the ELAgNPs did not show any activity up to 100 ppm against *B. subtilis*. The ELAgNPs showed the maximum inhibitory activity against *B. subtilis* in contrast to SN at 250 ppm and 500 ppm with a ZOI of 20.85 ± 0.86 mm and 20.97 ± 0.24 mm, respectively, whereas SN exhibited a ZOI of 16.83 ± 0.42 mm at 500 ppm (Supplementary Table S1). After 24 h of incubation, SN showed a ZOI of 21.42 ± 0.58 mm whereas the ELAgNPs exhibited the maximum inhibition of 21.98 ± 0.59 mm against *B. subtilis* at 500 ppm [Figure 5i]. Our results indicated that the ELAgNPs showed greater antibacterial activity against *B. subtilis* compared with SN. Moreover, with the increasing exposure time, the antibacterial activity of synthesized AgNPs increased [Figure 6, panel i, (a, b)].

The ELAgNPs exhibited a ZOI against *E. coli* of 15.31 ± 0.31 mm and 16.46 ± 0.22 mm at 500 ppm after 12 h and 24 h of incubation, respectively (Supplementary Table S1). No ZOI was observed in the case of ELExt and CAgNPs [Figure 5ii]. It was found that the ELAgNPs showed greater antibacterial efficacy against *B. subtilis* followed by *E. coli* [Figure 6, panel ii, (a, b)]. 

Our results are in agreement with previous reports showing green AgNPs to be more effective against (gram +ve) *B. subtilis* than against (gram −ve) *E. coli* [40,41]. Moreover, SN exhibited a larger ZOI compared with green synthesized AgNPs [42]. Our results are in consonance with a report exhibiting the relatively high antimicrobial potential of biogenic AgNPs in contrast with chemically synthesized AgNPs [43]. The antibacterial activity of AgNPs could be the sum of probable distinct mechanisms of action. These particles restrict enzyme activity by binding to the cell membrane, then destabilize it and ultimately lead to cell death by cytotoxicity, genotoxicity, and DNA damage [44]. Various investigations are still going on to decipher their mechanism of action. 

### 3.3. Antioxidant Activity of ELAgNPs

**Figure 7:**
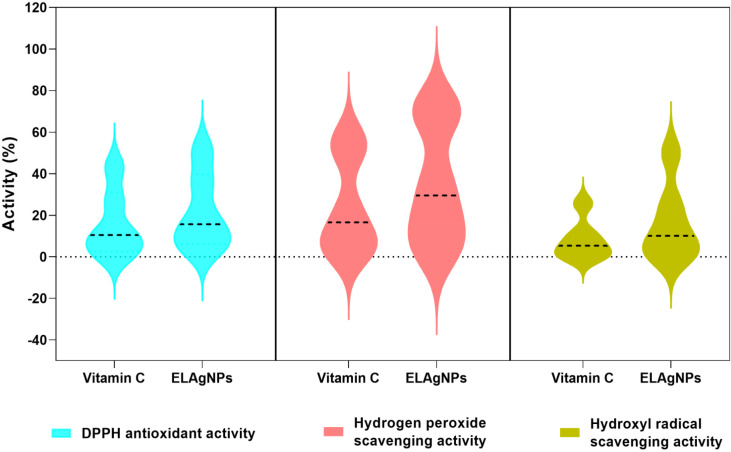
Grouped violin plot of antioxidant and scavenging activity (%) of green synthesized ELAgNPs with standard vitamin C.

The obtained results confirmed the antioxidant and scavenging potential of ELAgNPs. The ELAgNPs exhibited 51.23 ± 0.34% DPPH antioxidant activity compared with vitamin C (43.21 ± 1.21%) activity at 500 ppm as shown in Table 1. In addition, the ELAgNPs exhibited a greater hydrogen peroxide scavenging activity of 72.17 ± 0.56% in comparison with 57.75 ± 1.23% of vitamin C at a concentration of 500 ppm. It was observed that the ELAgNPs have a strong hydroxyl radical scavenging activity of 49.98 ± 0.56% compared with 25.67 ± 0.34% of vitamin C (Table 1) (Figure 7). The greater free radical scavenging property of ELAgNPs could be due to phyto-moieties such as anthocyanins, glycosides, and phenolic compounds present on their surface during bio-fabrication and stabilization. As depicted in the grouped violin plot of these activities, the ELAgNPs exhibited remarkable scavenging activity in contrast with standard vitamin C at all tested concentrations. The median activity percentile values of the ELAgNPs were higher than the standard and were distributed towards the median range as shown in Figure 7. Our results are in concurrence with the aqueous leaf extract of *Cestrum nocturnum*-based AgNPs which exhibited approximately 30% DPPH antioxidant activity, 20% hydroxyl radical scavenging activity, and 46% hydrogen peroxide scavenging activity with similar trends with reference to standard vitamin C [27].

### 3.4. Toxicity of ELAgNPs against VERO Cell Line

Cytotoxicity studies are crucial to understanding the effectiveness and safety of a drug/agent in a biological system. It is essential to find out whether the drug is toxic or not for targeted cells/tissues without affecting the healthy cells. In the present study, the cytotoxicity of the ELAgNPs was checked against Vero, the most practiced continuous mammalian cell line in the production of viral vaccines. For the past 40 years, this cell line has been used for the development of several vaccines against rabies, polio, smallpox, influenza, dengue, and Ebola. Recently, Vero cells have been used as a manufacturing platform for the COVID-19 vaccine as they are prone to MERS-CoV, SARS-CoV and SARS-CoV-2 [45]. The results clearly showed that the toxicity of ELAgNPs, CAgNPs and SN towards a cell line was significantly dose-dependent and caused a direct increase in cell mortality, whereas ELExt did not show any cytotoxic effect. 

**Figure 8:**
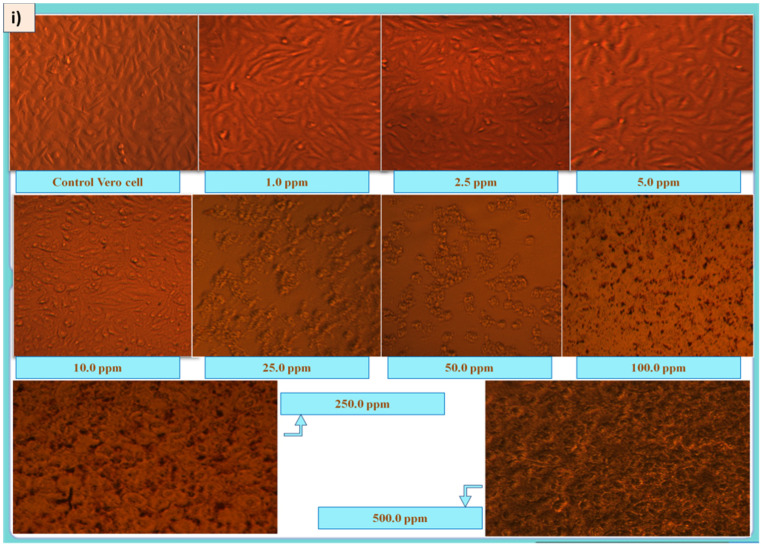

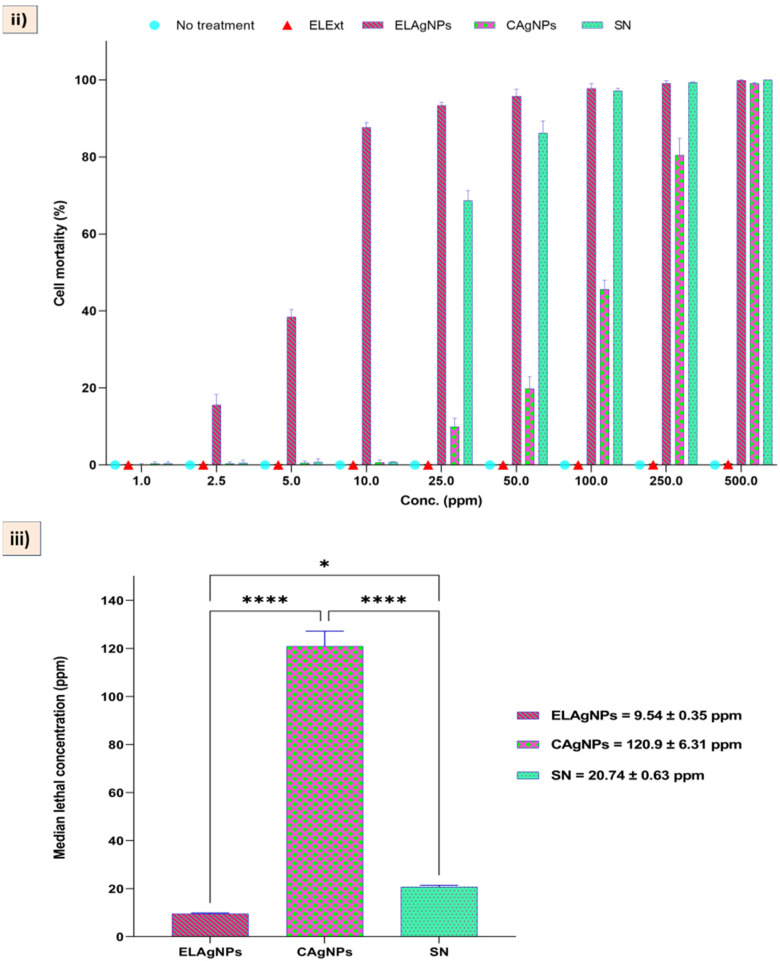
(**i**) Morphological changes in Vero cells due to ELAgNPs exposure at different concentrations, (data are presented as mean ± SD of three replicates). (**ii**) Cell mortality (%) of ELExt, ELAgNPs, CAgNPs, and SN after 48 h of incubation. (**iii**) LC_50_ of ELAgNPs, CAgNPs, and SN (where **** = *p* < 0.0001 and * = 0.01 < *p* < 0.05).

A minimum cell mortality of 0.17 ± 0.22%, 0.36 ± 0.43%, and 0.39 ± 0.42% was obtained for ELAgNPs, CAgNPs, and SN at 1 ppm, respectively, whereas a maximum cell mortality of 99.10 ± 0.72%, 99.05 ± 0.30%, and 99.29 ± 0.19% was obtained for ELAgNPs, CAgNPs, and SN at 250 ppm, 500 ppm, and 250 ppm, respectively. ELExt showed a maximum cell mortality of approximately 2% at 500 ppm [Figure 8i,ii)]. The median lethal concentration was found to be 9.54 ± 0.35 ppm, 120.9 ± 6.31 ppm, and 20.74 ± 0.63 ppm for ELAgNPs, CAgNPs, and SN, respectively. The ordinary one-way ANOVA results showed an extremely significant decrease in cell viability after 48 h of incubation in contrast with the cell control (*p* < 0.0001, and 0.01 < *p* < 0.05, α = 0.05), while an LC50 for the ELExt could not be calculated because of a very low cell mortality rate even at higher concentrations [Figure 8iii]. 

Our outcomes are in concurrence with the research of Saleh et al. (2019) [46] exhibiting that AgNPs did not affect the cell viability of Vero cells at lower concentrations of 31.25, 62.50, 125.00, and 250.00 ppm. However, a complete loss of viability was reported at higher concentrations of 500 ppm and 1000 ppm. In the case of aqueous flower extract of *Musa acuminate*-based AgNPs, IC_50_ of 55ppm was reported against Vero cells [47]. AgNPs exhibited cytotoxicity via interaction with functional groups of cellular proteins, entering the cells by endocytosis, followed by their retention by P-glycoproteins [48].

## 4. Conclusions

The current study lays emphasis on this proposed environment-friendly, inexpensive, undemanding, non-pathogenic, economical and speedy approach for the bio-fabrication of AgNPs using the aqueous leaf extract of *E. ferox* (Makhana), which is considered to be biowaste except its few applications in Chinese medicine. This is the first report on the synthesis of ELAgNPs and their efficaciousness as bactericidal, radical scavenging and cytotoxic agents. Phyto-constituents present in the ELExt not only acted as reducing agents but also as stabilizing and coating agents for the synthesis of small and stable ELAgNPs without the use of any accelerators and harmful chemicals. It is illustrious that these green synthesized ELAgNPs performed better compared with chemically synthesized CAgNPs in terms of elevated biological potentialities in a dose-dependent manner, including antibacterial activity against *B. subtilis* and *E. coli,* and cytotoxicity against Vero cell lines. They can be used as efficient diagnostic and therapeutic agents against microbial infections either alone or in combination with drugs in current COVID-compromised situations. They can also be helpful for understanding the effectiveness and safety of a drug/agent in a biological system via cytotoxicity analysis. In conclusion, the multifaceted functionality of biogenic AgNPs makes the green approach more promising. In future, we would like to explore the anticancerous properties of ELAgNPs as this showed cytotoxicity in a dose-dependent manner.

## Figures and Tables

**Table 1:** Antioxidant activity of ELAgNPs.

Concentration (ppm)	DPPH Antioxidant Activity (%)		Hydrogen Peroxide Scavenging Activity (%)		Hydroxyl Radical Scavenging Activity (%)	
	Vitamin C	ELAgNPs	Vitamin C	ELAgNPs	Vitamin C	ELAgNPs
1	0.82 ± 0.43	3.09 ± 0.38	0.92 ± 0.88	1.23 ± 0.11	0.00 ± 0.00	0.00 ± 0.00
10	3.11 ± 0.78	7.13 ± 0.92	5.77 ± 0.56	10.09 ± 0.83	0.00 ± 0.00	2.12 ± 1.02
50	9.23 ± 1.02	11.89 ± 1.32	10.37 ± 0.78	21.67 ± 0.34	3.78 ± 0.45	6.17 ± 1.15
100	11.76 ± 0.67	19.45 ± 0.13	22.81 ± 0.92	37.34 ± 0.23	6.89 ± 0.48	14.02 ± 1.03
250	26.89 ± 0.89	35.72 ± 0.22	49.93 ± 0.67	68.13 ± 0.78	11.42 ± 0.86	25.38 ± 0.23
500	43.21 ± 1.21	51.23 ± 0.34	57.75 ± 1.23	72.17 ± 0.56	25.67 ± 0.34	49.98 ± 0.56

## Data Availability

Data are available on request.

## Supplementary Materials

The following supporting information can be downloaded at: https://www.mdpi.com/article/10.3390/plants11202766/s1, Table S1. Antibacterial activity of green synthesized AgNPs against *B. subtilis* and *E. coli*. Table S2. Cytotoxic effect of green synthesized ELAgNPs against the Vero cell line.
