# Correction: Devi et al. Bio-Fabrication of *Euryale ferox* (Makhana) Leaf Silver Nanoparticles and Their Antibacterial, Antioxidant and Cytotoxic Potential. *Plants* 2022, *11*, 2766

**DOI:** 10.3390/plants15132050

**Published:** 2026-07-02

**Authors:** Nisha Devi, Kanika Rani, Pushpa Kharb, Prashant Kaushik

**Affiliations:** 1 Department of Molecular Biology, Biotechnology and Bioinformatics, Chaudhary Charan Singh Haryana Agricultural University, Hisar 125004, India; nishu.sharma57605@gmail.com (N.D.); kvats54@gmail.com (K.R.); 2 Center for Bio-Nanotechnology, Chaudhary Charan Singh Haryana Agricultural University, Hisar 125004, India; 3 Kikugawa Research Station, Yokohama Ueki, Kikugawa 439-0031, Japan

In the publication [1], there was an error regarding the affiliation of Prashant Kaushik. The affiliation “Instituto de Conservación y Mejora de la Agrodiversidad Valenciana, Universitat Politècnica de València, 46022 Valencia, Spain” has been removed, as per the institution’s request. The authors state that the scientific conclusions are unaffected. This correction was approved by the Academic Editor. The original publication has also been updated.
