# Supplementary material for: Bio-Fabrication of Euryale ferox (Makhana) Leaf Silver Nanoparticles and Their Antibacterial, Antioxidant and Cytotoxic Potential
Source: Plants (Basel). 2022 Oct 19;11(20):2766. doi: 10.3390/plants11202766 (PMC9612292; doi:10.3390/plants11202766)
Supplement: Supplementary file 1 [file plants-11-02766-s001.zip › plants-1962246-supplementary.pdf]

Supplementary file

Table S1. Antibacterial activity of green synthesized AgNPs against *B. subtilis*, and *E. coli*

**Treatment**	**Conc. (ppm)**	***B. subtilis***		***E. coli***	
		**ZOI after incubation (h)**		**ZOI after incubation (h)**	
		**12**	**24**	**12**	**24**
Media control		0.00±0.00	0.00±0.00	0.00±0.00	0.00±0.00
ELAgNPs	1	-	-	-	-
	10	-	-	-	-
	50	-	-	-	-
	100	-	15.27±0.43	-	-
	250	20.85± 0.86	21.03±0.53	13.56±0.59	15.23±0.42
	500	20.97± 0.24	21.98±0.59	15.31±0.31	16.46±0.22
CAgNPs	1	-	-	-	-
	10	-	-	-	-
	50	-	-	-	-
	100	-	-	-	-
	250	-	-	-	-
	500	-	-	-	-
SN	1	-	-	-	-
	10	-	11.20 ±0.71	-	
	50	-	18.47 ±0.83	14.11±0.43	14.86±0.36
	100	13.85 ± 0.35	20.12 ±0.76	14.83±0.58	15.07±0.48
	250	16.27 ± 0.73	21.11 ±0.62	15.45±0.63	16.93±0.89
	500	16.83± 0.42	21.42±0.58	16.10±0.41	17.65±0.45
ELExt	1	-	-	-	-
	10	-	-	-	-
	50	-	-	-	-
	100	-	-	-	-
	250	-	-	-	-
	500	-	-	-	-

Note: - indicates no ZOI observed. Data is presented as Mean±SD of four replicates.

(where, ELAgNPs= aqueous leaf extract of *E. ferox* based AgNPs, CAgNPs= commercial AgNPs, SN= $AgNO_3$ solution, and ELExt= aqueous leaf extract of *E. ferox*)

Table S2. Cytotoxic effect of green synthesized ELAgNPs against Vero cell line

Treatment	Conc. (ppm)	OD	Cell mortality (%)	LC_{50} (ppm)	P value (α=0.05, 95% CI)
Cell control (No treatment)		1.19±0.01	0.00±0.51	–	–
	1.0	1.19±0.00	0.14±0.17		
	2.5	1.19±0.00	0.31±0.30		
	5.0	1.18±0.01	0.53±0.46		
	10.0	1.18±0.00	0.53±0.35		
ELExt	25.0	1.18±0.01	1.04±0.56	–	–
	50.0	1.17±0.00	1.32±0.39		
	100.0	1.18±0.00	0.87±0.05		
	250.0	1.17±0.01	1.57±0.56		
	500.0	1.18±0.01	1.02±1.04		
	1.0	1.19±0.00	0.17±0.22		
	2.5	1.00±0.03	15.60±2.62		
	5.0	0.73±0.02	38.43±1.93		(****) $p<0.0001^{a-b}$
	10.0	0.15±0.02	87.68±1.27		
ELAgNPs[a]	25.0	0.08±0.01	93.33±0.85	9.54±0.35	(*) $0.01<p<0.05^{a-c}$
	50.0	0.05±0.02	95.77±1.72		
	100.0	0.03±0.02	97.73±1.29		
	250.0	0.01±0.01	99.10±0.72		
	500.0	0.00±0.00	99.83±0.22		
	1.0	1.19±0.01	0.36±0.43	120.9±6.31	
	2.5	1.19±0.00	0.39±0.38		(****) $p<0.0001^{b-a}$
	5.0	1.18±0.01	0.50±0.50		
	10.0	1.18±0.01	0.70±0.64		
CAgNPs[b]	25.0	1.07±0.03	9.94±2.19		(****) $p<0.0001^{b-c}$
	50.0	0.95±0.04	19.80±3.04		
	100.0	0.65±0.03	45.60±2.35		
	250.0	0.23±0.05	80.48±4.33		
	500.0	0.01±0.00	99.05±0.30		
	1.0	1.19±0.01	0.39±0.42		(*) $0.01<p<0.05^{c-a}$
	2.5	1.18±0.01	0.53±0.71		
	5.0	1.18±0.01	0.78±0.85		(****) $p<0.0001^{c-b}$
	10.0	1.18±0.00	0.76±0.08		
SN[c]	25.0	0.37±0.03	68.63±2.61		
	50.0	0.16±0.04	86.16±3.23	20.74±0.63	
	100.0	0.03±0.01	97.17±0.61		
	250.0	0.01±0.00	99.29±0.19		
	500.0	0.00±0.00	99.99±0.02		

Note: − indicates not applicable. Data is presented as Mean±SD of three replicates. [a-c] denote multiple comparisons b/w treatments and ****=extremely significant, and *= significant

(where, ELExt= aqueous leaf extract of *E. ferox*, ELAgNPs= aqueous leaf extract of *E. ferox* based AgNPs, CAgNPs= commercial AgNPs, and SN= $AgNO_3$ solution)
